# New and known type 2 diabetes as coronary heart disease equivalent: results from 7.6 year follow up in a middle east population

**DOI:** 10.1186/1475-2840-9-84

**Published:** 2010-12-04

**Authors:** Farzad Hadaegh, Nooshin Fahimfar, Davood Khalili, Farhad Sheikholeslami, Fereidoun Azizi

**Affiliations:** 1 Prevention of Metabolic Disorders Research Center, Research Institute for Endocrine Sciences, Shahid Beheshti University of Medical Sciences, Parvaneh St., Yaman St., Velenjak, Tehran, Iran; 2 Department of Epidemiology, School of Public Health, Shahid Beheshti University of Medical Sciences, Daneshjoo Blvd, Tehran, Iran; 3 Endocrine Research Center, Research Institute for Endocrine Sciences, Shahid Beheshti University of Medical Sciences, Parvaneh St., Yaman St., Velenjak, Tehran, Iran

## Abstract

**Background:**

To investigate whether the known diabetes mellitus (KDM) or newly diagnosed diabetes mellitus (NDM) could be regarded as a coronary heart disease (CHD) risk equivalent among a relatively young Middle East population with high prevalence of diabetes mellitus (DM).

**Methods:**

A population based cohort study of 2267 men and 2931 women, aged ≥ 30 years. Prior CHD was defined as self-reported or ECG positive CHD at baseline, KDM as subjects using any kind of glucose-lowering medications and NDM according to fasting plasma glucose and 2-h postchallenge glycemia.

Participants were categorized to six groups according to the presence of known or newly diagnosed DM and CHD at baseline (DM-/CHD-, DM-/CHD+, NDM+/CHD-, NDM+/CHD+, KDM+/CHD-, KDM+/CHD+) and Cox regression analysis were used to estimate the hazard ratio (HR) of CHD events for these DM/CHD groups, given DM-/CHD-as the reference.

**Results:**

During 7.6-year follow up, 358 CHD events occurred. After controlling traditional risk factors, HRs of CHD events for DM-/CHD+ group were 2.1 (95% CI: 1.4-3.1) and 5.2 (3.2-8.3) in men and women respectively. Corresponding HRs for NDM+/CHD-were 1.7 (1.1-2.7) and 3.1 (1.8-5.6) and for KDM+/CHD-were 1.7 (0.9-3.3) and 6.2 (3.6-10.6) in men and women respectively. The HRs for NDM+/CHD+ and KDM+/CHD+ groups (i.e. participants with history of both diabetes and CHD) were 6.4 (3.2-12.9) and 8.0 (4.3-14.8) in women and 3.2 (1.9-5.6) and 4.2 (2.2-7.8) in men, respectively.

The hazard of CHD events did not differ between KDM+/CHD-and DM-/CHD+ in both genders using paired homogeneity test, however the HR for NDM+/CHD-was marginally lower than the HR for DM-/CHD+ in women (*p *= 0.085).

**Conclusions:**

KDM patients in both genders and NDM especially in men exhibited a CHD risk comparable to nondiabetics with a prior CHD, furthermore diabetic subjects with prior CHD had the worst prognosis, by far more harmful in women than men; reinforcing the urgent need for intensive care and prophylactic treatment for cardiovascular diseases.

## Background

The most important cause of mortality in diabetic patients is cardiovascular disease (CVD) [1] and observational studies have shown that diabetes mellitus (DM) increases risk of CVD nearly two to three folds [1,2]. In addition, some cohort studies conducted in American and European populations have suggested that rates for coronary heart disease (CHD) events are equivalent to both individuals with prior CHD and diabetic subjects without prior CHD [1-4]. Therefore, the American Diabetes Association recommended that, in secondary prevention, the diabetic patients should be treated for the same lipid and blood pressure targets as subjects with previous myocardial infarction [5]. Some other studies have found that cardiovascular risk are lower in subjects with diabetes but without CHD, than in persons with CHD and without diabetes [6-8]; however a few studies have also shown contradictory results [2,3]. Importantly, it is not clear whether these findings can be generalized to other populations because relative mortality risks may differ with ethnicity [9-11].

It is estimated that developing countries in Asia and in the Middle East, particularly in Persian Gulf states, will have the largest increases in the prevalence of diabetes by 2030, which is related to major shift in life style and nutrition transition in these countries [12-17]. Nevertheless, to our best knowledge, there is no study to show the equivalency of diabetes and prior CHD for risk of CHD in this region.

The prevalence of Type 2 diabetes is reported to be more than 14% in Tehran, Iran, with an estimated incidence of new cases in about 1% of the population per year [14,15]. There is a high prevalence of CHD in this area too [18] and it has been shown that the Iranian population with diabetes has a high risk for CVD, independent of traditional risk factors [19]. Hence, we compared the risk of CHD events among diabetic participants without prior CHD to that of participants diagnosed as having CHD but not diabetes in the framework of the Tehran Lipid and Glucose Study (TLGS) which is a population based study conducted on a representative population of the capital city, Tehran [20]. Furthermore, we analyzed the results separately for newly diagnosed DM and known DM, and in addition to a prior history of CHD, we applied electrocardiographic criteria for the presence of CHD at baseline.

## Methods

### Study population

**Figure 1:**
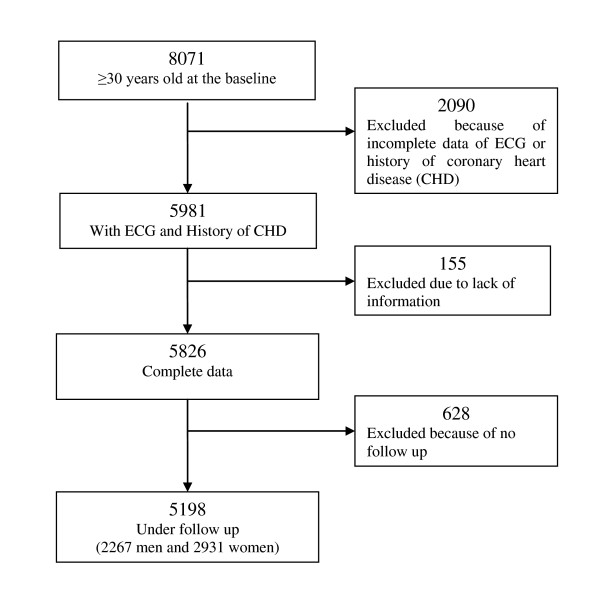
**Study participants' entry**.

The TLGS is a prospective population-based study to determine the risk factors for non-communicable diseases among a representative urban population of Tehran [20]. The sampling method has been described elsewhere [20]. Briefly, a total of 15005 individuals aged 3 years and over who were residences of district No.13 of Tehran were selected using multistage cluster random sampling method. Subjects were categorized into the cohort and intervention groups, the latter to be educated for implementation of life style changes [20]. From among 8071 individuals, aged ≥ 30 years, who participated in the first phase of TLGS (February 1999 to August 2001), 5981 subjects had complete data of electrocardiogram (ECG) and history of CHD. Excluding participants with other missing data (n = 155) resulted in 5826 subjects at baseline; of those we followed 5198 (64.4% of total) until 20 March 2008 with a median follow up of 7.6 years (Figure 1). Written informed consent was obtained from all subjects and the ethical committee of Research Institute for Endocrine Sciences approved this study.

### Clinical and laboratory measurements at enrolment

The information was collected during a personal interview, and completion of a questionnaire for demographic factors, medical history, medication use and smoking. Physical examination for blood pressure and anthropometrical measurements was performed. Blood pressure was measured twice in a seated position after 15 min resting using a standard mercury sphygmomanometer and the mean of the two measurements was considered as the subject's blood pressure. Body mass index (BMI) was calculated as weight (kg) divided by the square of height (m^2^).

Two trained technicians recorded a 12-lead ECG according to the standard recording protocol developed by the School of Public Health, the University of Minnesota using a PC-ECG 1200 machine [21]. Two qualified physicians coded the ECGs in parallel according to the Minnesota codes using a measuring loupe specially manufactured by University of Minnesota [21]. Then, for assurance of quality a third qualified physician recoded 10% of ECGs. All the data were double-checked.

Fasting plasma glucose (FPG) and lipid profiles were tested after 12-14 hours overnight fasting. Standard oral glucose tolerance test (OGTT) was performed in participants without treated diabetes. All analyses were done at the TLGS research laboratory on the day of collection. Plasma glucose, serum total cholesterol, high-density lipoprotein cholesterol (HDL-C) and triglyceride (TG) levels were measured by using previously reported methods [20].

### Definition of terms

Family history of premature CVD reflected prior diagnosis of CVD in female first-degree relatives, aged less than 65 years or male first-degree relatives under the age of 55 years old.

Smoking was defined as history of current smoking (including daily or occasional). Newly diagnosed DM (NDM) was defined as individuals with FPG ≥ 7.0 mmol/L (126 mg/dl) or 2-h postchallenge glycemia (2hPG) ≥ 11.1 mmol/L (200 mg/dl) without any history of glucose-lowering medications and Known DM (KDM) was defined as subjects who have been treated with any kind of glucose-lowering medications.

### Prevalent CHD at enrolment

Prevalent (or prior) CHD was defined as self-reported CHD or ECG positive CHD. Self-reported CHD was defined as a positive answer to the question as to "whether the subject has ever had a prior diagnosis of CHD by a physician". Electrocardiogram positive CHD was defined according to the Whitehall criteria [22] which categorize subjects into three groups, non CHD, probable CHD (any Minnesota codes of 1.1.1 through 1.1.7and 1.2.1 through 1.2.8) and possible CHD (codes of 1.3.1 through 1.3.6, 4.1.1 through 4.4, 5.1 through 5.3 or 7.1.1 through 7.1.2). In our analysis, we considered both probable and possible CHD as a single definition of ECG-positive CHD.

We divided our cohort into six groups based on their clinical status at the baseline examination:

- Subjects without newly diagnosed or known DM and without prior CHD (DM-/CHD-)

- Subjects without newly diagnosed or known DM but with prior CHD (DM-/CHD+)

- Subjects with NDM but without prior CHD (NDM+/CHD-)

- Subjects with NDM and with prior CHD (NDM+/CHD+)

- Subjects with KDM but without prior CHD (KDM+/CHD-)

- Subjects with KDM and with prior CHD (KDM+/CHD+)

### Definition of CHD outcome

Each TLGS participant is under continuous surveillance for any medical event leading to hospitalization during the previous year by telephone call and he/she is questioned by a trained nurse regarding any medical conditions. If a related event has occurred, a trained physician collects complementary data during a home visit, and when deemed necessary, a visit to the respective hospital for collecting data from the participant's medical files was done. In the case of mortality, data are collected from the hospital or death certificate by an authenticated local physician. Collected data are evaluated by an outcome committee consisting of a principal investigator, an internist, an endocrinologist, a cardiologist, an epidemiologist and the physician who collected the outcome data; other experts are invited for evaluation of non-communicable disorders as needed. Specific outcome for every event is assigned according to ICD-10 criteria and American Heart Association classification for cardiovascular events [20].

In the current study, CHD events as outcomes included cases of definite myocardial infarction (MI) diagnosed by electrocardiogram (ECG) and biomarkers, probable MI (positive ECG findings plus cardiac symptoms or signs but biomarkers showing negative or equivocal results), unstable angina pectoris (new cardiac symptoms or changing symptom patterns and positive ECG findings with normal biomarkers), angiographic proven CHD and CHD death.

### Statistics

The results for continues variables are given as mean (± SD) and for categorical variables as percentages. Comparisons of the baseline characteristics among DM/CHD groups were done using ANOVA or χ2 test and we used t-test or χ2 test to compare any group with the DM-/CHD+ group considering Bonferroni correction for multiple comparisons.

Cox proportional hazard regression models were used to estimate the hazard ratio (HR) of CHD events for DM/CHD groups (DM-/CHD+, NDM+/CHD-, NDM+/CHD+, KDM+/CHD-, KDM+/CHD+) in both genders, given DM-/CHD-as the reference group. Follow up duration was defined as the period between the entrance to study and the end point; end point was considered as the first CHD event and participants were censored at non-CHD death or end of follow-up. Analyses were done with adjustment for age alone and for age with other possible risk factors including systolic blood pressure, body mass index (BMI), total cholesterol, TG/HDL-C [23], family history of premature CVD, smoking and being in the intervention group. The proportional hazards assumption in the Cox model was assessed using log minus log plot of survival and Schoenfeld residual test. All proportional hazards assumptions were generally appropriate (p-value for global test of proportional hazards assumption was > 0.1 in men and women).

A paired homogeneity test, which is a Wald test of the linear hypothesis of the Cox model regression coefficients, was performed to test the null hypothesis that the hazard ratio for NDM+/CHD-and KDM+/CHD-was equal to that for DM-/CHD+.

Statistical analyses were performed using SPSS for windows version 15 and STATA version 10, and p-values less than 0.05 were considered statistically significant. All analyses were separated by gender regarding significant interaction between gender and some of the DM/CHD categories to predict CVD events.

## Results

In comparison to men who were not included in the study, those who were had less history of smoking (27% vs. 31.5%). Women included were younger (46.9 vs. 47.8 years), had lower systolic blood pressure (122 vs. 124 mmHg) and higher BMI (28.6 vs. 28.1 kg/m2) than those not included (all p < 0.05).

Baseline characteristics for all groups are shown in Table 1 and 2. Compared to the DM-/CHD+ group, NDM+/CHD-had higher TG/HDL-C and BMI values in men and higher TG/HDL-C, BMI and systolic blood pressure values in women; also KDM+/CHD-had higher systolic blood pressure and TG/HDL-C in women.

**Table 1:** General characteristics of men according to DM and prior CHD status at baseline ^a^

	DM/CHD group					
	
Variable^b^	DM-/CHD-	DM-/CHD+	NDM+/CHD-	NDM+/CHD+	KDM+/CHD-	KDM+/CHD+
**number**	1726	228	152	52	69	40
**Age (years)**	46.1 (12.1)*	56.9(12.2)	55.7(11.6)	60.8(11.6)	59.5(9.2)	63.3(8.1)*
**SBP (mm Hg)**	119.3 (17.0)*	131.8(23.0)	132.1(20.5)	144.1(32.0)*	132.4(20.4)	139.9(27.6)
**DBP (mm Hg)**	78.0 (10.7)*	80.8(12.8)	82.0(12.5)	84.3(15.7)	80.5(10.3)	81.65(13.3)
**TC (mmol/L)**	5.39 (1.07)*	5.78(1.16)	5.86(1.21)	5.83(1.08)	5.60(1.26)	5.61(1.07)
**TG/HDL-C**	5.3(4.5)*	6.1(4.6)	7.2(4.6)*	7.5(7.0)	7.5(6.9)	4.8(3.2)
**BMI (kg/m²)**	25.9 (3.8)*	27.0(3.7)	28.0(3.9)*	27.6(3.7)	27.6(4.0)	26.1(3.4)
**Smoking (%)**	29.0	21.1	20.4	32.7	20.3	17.5
**FH of CVD (%)**	14.8	18.4	13.2	13.5	11.6	12.5
**Intervention (%)**	38.0	36.8	38.2	40.4	58.0*	32.5

^a ^Data have been shown as mean (± sd) or percent.

^b ^In all variables except "FH of CVD" difference among categories is significant (P < 0.05 according to ANOVA or χ2 test.)

*Difference with "Any DM-/CHD+" group is significant at the level of P < 0.01 (according to t-test or χ2 test and considering Bonferroni correction for multiple comparisons.).

DM: diabetes mellitus, CHD: coronary heart disease, NDM: newly diagnosed DM, KDM: known DM, SBP: systolic blood pressure, DBP: diastolic blood pressure, TC: total cholesterol, TG/HDL-C: triglyceride/HDL cholesterol, BMI: body mass index, FH of CVD: family history of premature cardiovascular disease, Intervention: being in intervention group.

**Table 2:** General characteristics of women according to DM and prior CHD status at baseline ^a^

	DM/CHD group					
	
Variable ^b^	DM-/CHD-	DM-CHD+	NDM+/CHD-	NDM+/CHD+	KDM+/CHD-	KDM+/CHD+
**number**	2184	285	210	56	138	58
**Age (years)**	44.6(10.7)*	53.0(11.7)	51.2(10.0)	57.2(8.3)*	55.4(9.6)	60.6(7.8)*
**SBP (mm Hg)**	118.1(17.6)*	129.3(22.9)	133.9(21.6)*	139.2(22.5)*	135.8(23.0)*	140.0(23.3)*
**DBP (mm Hg)**	78.1(10.0)*	82.4(12.5)	84.5(11.8)	85.6(9.7)	82.6(10.5)	81.5(12.2)
**TC (mmol/L)**	5.61 (1.15)*	6.14(1.26)	6.24(1.25)	6.53(1.57)	6.34(1.32)	6.87(1.33)*
**TG/HDL-C**	4.1(3.4)*	4.9(3.9)	6.8(5.4)*	5.8(3.8)	5.9(4.4)*	6.4(4.4)*
**BMI (kg/m²)**	28.2(4.6)*	29.3(4.8)	30.6(4.9)*	30.1(5.0)	29.2(4.8)	29.4(5.2)
**Smoking (%)**	3.9	3.9	3.8	0.0	2.2	3.4
**FH of CVD (%)**	17.5	22.1	26.2	16.1	23.2	25.9
**Intervention (%)**	39.1	34.7	41.4	41.1	37.0	46.6

^a ^Data have been shown as mean (± sd) or percent.

^b ^In all variables except smoking and intervention difference among categories is significant (P < 0.05 according to ANOVA or χ2 test.)

*Difference with "Any DM-/CHD+" group is significant at the level of P < 0.01 (according to t-test or χ2 test and considering Bonferroni correction for multiple comparisons.).

DM: diabetes mellitus, CHD: coronary heart disease, NDM: newly diagnosed DM, KDM: known DM, SBP: systolic blood pressure, DBP: diastolic blood pressure, TC: total cholesterol, TG/HDL-C: triglyceride/HDL cholesterol, BMI: body mass index, FH of CVD: family history of premature cardiovascular disease, Intervention: being in intervention group.

Overall, 212 (9.4%) men and 146 (5%) women had CHD events during 16033 and 21433 person-years of follow up; the corresponding incidence density of CHD event were 13.2 and 6.8 per 1000 person-years in men and women respectively. In men, 46 (20.2%) of DM-/CHD+, 26 (17.1%) of NDM+/CHD-and 11(15.9%) of KDM+/CHD-individuals experienced CHD events; these values in women were 39 (13.7%), 19 (9.0%) and 25 (18.1%) respectively.

**Figure 2:**
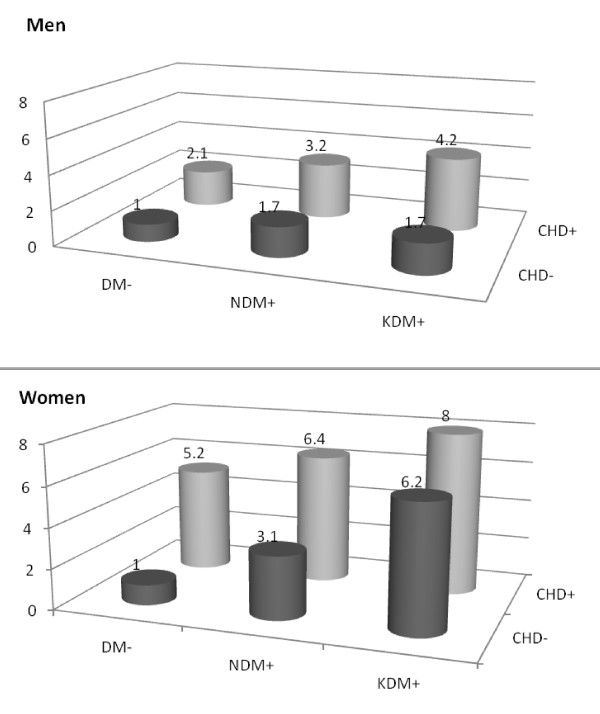
**Hazard ratio of CHD event according to baseline DM and prior CHD status**. Legend: DM: diabetes mellitus, CHD: coronary heart disease, NDM: newly diagnosed DM, KDM: known DM.

Table 3 and 4 and Figure 2 highlight the HR of coronary heart disease in relation with DM and prior CHD in men and women. Age adjusted analysis revealed notably higher risks in DM-/CHD+, NDM+/CHD-and KDM+/CHD-groups compared with DM-/CHD-in both genders. However, in multivariate analysis, DM-/CHD+ and NDM+/CHD-in both genders and KDM+/CHD-only in women remained as significant predictors of CHD events. The risks of patients with DM or CHD were higher in women than men (in all of the DM/CHD categories) and *p-*value for the effect modification by gender reached the significant level of 0.05 in DM-/CHD+ and KDM+/CHD-groups (*p *= 0.002 for both). Hazard ratios of CHD events were 2.1 (95%CI: 1.4-3.1), 1.7 (1.1-2.7) and 1.7 (0.9-3.3) for DM-/CHD+, NDM+/CHD-and KDM+/CHD-respectively in men, values which were 5.2 (3.2-8.3), 3.1 (1.8-5.6) and 6.2 (3.6-10.6) in women.

**Table 3:** Hazard ratio of CHD event in men according to baseline DM and prior CHD status

	CHD event NO. (rate)^a^	Age adjusted HR	p-value	Multivariate^b ^adjusted HR	p-value
**DM-/CHD-**	98 (7.8)	Ref		Ref	
**DM-/CHD+**	46 (30.5)	2.8(1.9-4.0)	< 0.001	2.1(1.4-3.1)	< 0.001
**NDM+/CHD-**	26 (25.5)	2.4(1.5-3.7)	< 0.001	1.7(1.1-2.7)*	0.020
**NDM+/CHD+**	19 (63.3)	5.3(3.2-8.9)	< 0.001	3.2(1.9-5.6)	< 0.001
**KDM+/CHD-**	11 (24.7)	2.2(1.1-4.1)	0.017	1.7(0.9-3.3)†	0.108
**KDM+/CHD+**	12 (55.5)	4.7(2.5-8.7)	< 0.001	4.2(2.2-7.8)	< 0.001

^a ^Incidence rate: number of cases per 1000 person-year.

^b ^Analyses were adjusted for age, systolic blood pressure, body mass index, cholesterol, triglyceride/HDL-cholesterol, family history of cardiovascular disease, smoking and intervention.

* The p-value to compare with the HR of DM-/CHD+ was 0.414, applying Wald test.

† The p-value to compare with the HR of DM-/CHD+ was 0.527, applying Wald test.

DM: diabetes mellitus, CHD: coronary heart disease, NDM: newly diagnosed DM, KDM: known DM, HR: hazard ratio.

**Table 4:** Hazard ratio of CHD event in women according to baseline DM and prior CHD status

	CHD event NO. (rate)^a^	Age adjusted HR	p-value	Multivariate^b ^adjusted HR	p-value
**DM-/CHD-**	35 (2.1)	Ref		Ref	
**DM-/CHD+**	39 (19.6)	6.0(3.7-9.6)	< 0.001	5.2(3.2-8.3)	< 0.001
**NDM+/CHD-**	19 (12.8)	4.4(2.5-7.7)	< 0.001	3.1(1.8-5.6)*	< 0.001
**NDM+/CHD+**	11 (30.4)	8.2(4.1-16.4)	< 0.001	6.4(3.2-12.9)	< 0.001
**KDM+/CHD-**	25 (26.5)	7.4(4.4-12.6)	< 0.001	6.2(3.6-10.6)†	< 0.001
**KDM+/CHD+**	17 (48.2)	11.3(6.1-20.8)	< 0.001	8.0(4.3-14.8)	< 0.001

^a ^Incidence rate: number of cases per 1000 person-year.

^b ^Analyses were adjusted for age, systolic blood pressure, body mass index, cholesterol, triglyceride/HDL-cholesterol, family history of cardiovascular disease, smoking and intervention.

* The p-value to compare with the HR of DM-/CHD+ was 0.085, applying Wald test.

† The p-value to compare with the HR of DM-/CHD+ was 0.475, applying Wald test.

DM: diabetes mellitus, CHD: coronary heart disease, NDM: newly diagnosed DM, KDM: known DM, HR: hazard ratio.

Considering both NDM and KDM in a DM+/CHD-group, the HR of this group was 1.73 (95%CI: 1.15-2.60) and 4.37 (2.74-6.97) compared to DM-/CHD-group in men and women, respectively.

Individuals with known or newly diagnosed DM and prior CHD demonstrated additive risks, i.e. HRs of 3.2 (95% CI: 1.9-5.6) and 4.2 (2.2-7.8) in men and 6.4 (3.2-12.9) and 8.0 (4.3-14.8) in women for NDM+/CHD+ and KDM+/CHD+ respectively.

Finally, as shown in Table 3 and 4, the results of the Wald test, used to compare the hazard ratios, highlighted that the risk of CHD events did not differ between KDM+/CHD-and DM-/CHD+ in both genders, however the risk of NDM+/CHD-was marginally lower than the HR of DM-/CHD+ in women (*p *= 0.085).

## Discussion

When history of CHD or ischemic ECG changes was used to define prior CHD, this study showed that Type 2 diabetes is a "CHD equivalent" in a 7.6-year follow-up of Iranian subjects. The risk of CHD associated with diabetes was relatively equal to that associated with prior CHD, each conferring a ≈2 and ≈ 4-fold increased risk of CHD in men and women, respectively. Our findings are consistent with the highly cited Haffner et al' paper, in which Finnish known Type 2 diabetic patients without prior myocardial infarction have as high a risk of myocardial infarction as nondiabetic patients with prior myocardial infarction [24]. Because the Haffner et al. study included only 69 DM-/CHD+ individuals, the power of the study to detect differences between the 2 groups was limited. Similarly, in a population based study in Denmark, diabetic patients requiring glucose-lowering agents exhibited a cardiovascular risk comparable to nondiabetic with a prior myocardial infarction, regardless of sex and diabetes type [25]. However, Our findings are in contrast with those of Lee et al, in their Atherosclerosis Risk in Communities study (ARIC), in which after adjustment for multiple baseline risk factors, patients who had a history of myocardial infarction without diabetes at baseline had a 1.9 times the risk of fatal CHD or nonfatal myocardial infarction, compared with diabetic patients without a prior history of myocardial infarction [26]. Furthermore, in the Nurses' Health Study [27] and Health professional Follow-up study [8], Type 2 diabetic patients without myocardial infarction had a lower risk of CHD compared with myocardial infarction patients without diabetes. Recently, data from the REACH registry do not fully support the concept that diabetes is a cardiovascular risk equivalent, and the rate of pooled CVD mortality and nonfatal myocardial infarction in nondiabetic subjects with previous CAD was more than 50% higher than that seen in diabetic patients with risk factors only [28]. It is important to note that, the studies addressing these issues have great differences in age distribution, designs, and definitions of the study populations that could explain the discrepancies with our findings.

Recently we found that diabetes, in particular the known cases, had higher risk more so in women than in men for CVD events [19]. In the present study, according to the diabetes status i.e. new vs. known cases, in women both the KDM+/CHD-and NDM+/CHD-groups had significant risk for incident CHD in comparison with the DM-/CHD-group (Risk factor adjusted HR ≈ 6 and ≈ 3 respectively, p < 0.001); however, in men only NDM+/CHD-, resulted in a significant risk, in comparison with DM-/CHD-(HR: 1.7, 95% CI: 1.07-2.69). The lack of association of KDM+/CHD-with incident CHD in men might be attributed to the limited statistical power.

As acknowledged by Gonzalez-Clemente et al, it would probably have been better to directly compare persons who were DM+/CHD-with those who were DM-/CHD+ [29], hence comparing directly the hazard ratios of the these groups using the paired homogeneity test highlighted no difference between the KDM+/CHD-and DM-/CHD+ groups in risk factor adjusted analysis in prediction of incident CHD. However, an important finding of the current study was that regarding NDM in women, the risk of incident CHD in multivariate analysis for participants with DM -/CHD+ was marginally higher than those with NDM+/CHD-, although this did not reach a significant level. Consistent with our findings, a similar impact of diabetes in those receiving glucose-lowering agents (i.e. KDM) vs. those with prior CHD in prediction of incident CHD was demonstrated in the Multiple Risk Factor Intervention Trial (MRFIT) of men, 35 to 57 years old [30]. Similarly, Carnethon et al in a longitudinal study of men and women aged ≥ 65 years highlighted that, CHD mortality risk was similar between participants with CHD alone vs. diabetes alone [31]. Recently Andrersson el, in a multivariate regression analyses showed that patients with diabetes and absence of significant coronary artery disease at angiography have impaired systolic longitudinal left ventricular function and a global diastolic dysfunction, which is likely to be associated with adverse prognosis [32].

In the present study, diabetic subjects with prior CHD had the worst prognosis, by far more harmful in women than men (multivariate adjusted HR 7.19 vs. 3.58, respectively) similar to the reports of other studies [2,27,29] highlighting the importance of secondary prevention in patients with both coexisting disorders, especially in women. Our data concerning higher risk of CVD in women than men with DM +/CHD+ is comparable with female sex predominance in acute coronary syndrome patients with diabetes from a hospital based study from Iran [33].

Our study has several strengths. This is the first population-based study in Caucasians of Middle East region, conducted to determine the equivalency of diabetes and prior CHD for risk of CHD event. We included newly diagnosed DM in our study based on the OGTT result; furthermore, the ischemic change of ECG was added for defining prevalent CHD. However, most of the studies on this issue, included diagnosis of CHD and diabetes based on data provided by the patients themselves, which may have led to misclassification of subjects in the various groups. Finally, our study also adjusted for major CVD risk factors in the statistical model.

## Limitations of the study

This study has important limitations that should be considered. First, we used positive history of CHD and the ECG-defined CHD as criteria to define prevalent CHD at baseline. The principal difference between these criteria might be caused by over reporting, non-Q-wave myocardial infarctions, or the interventions implemented to alleviate blockage of the coronary arteries [22]; unfortunately, at baseline the questionnaire of TLGS did not include questions about use of thrombolytic therapy, coronary artery bypass surgery, or percutaneous transluminal coronary angioplasty. Furthermore, population-based studies have found self-reported MI, CHD and stroke to be moderately or highly accurate in determining disease status [34]. Second, we defined newly diagnosed DM with a single OGTT at baseline as suggested for epidemiological studies; however, this might lead to attenuation of the association between newly diagnosed DM and cardiovascular events, resulting in an underestimation of the risk in this group. Third, the duration of diabetes could be a factor that explains the conflicting results in studies aiming to determine diabetes as a CHD risk equivalent, since longer duration of diabetes is associated with an increased risk of CVD [29]. Recently, Dagenais et al in the Quebec Cardiovascular Study emphasized finding that compared to men with incident CVD, men with incident diabetes had a lower risk for CVD mortality during the first 5 years after the diagnosis of diabetes, but subsequently there was no difference between the 2 groups for cardiovascular and total mortality [35]. Finally, our population was selected from middle-aged Middle East Caucasians and therefore we cannot make inferences beyond a similar group.

## Conclusions

The current study highlighted the finding that known diabetic patients in both genders and newly diagnosed diabetes especially in men, exhibited a CHD risk comparable to nondiabetics with a prior CHD, regardless of risk factors, furthermore diabetic subjects with prior CHD had the worst prognosis, by far more harmful in women than men; reinforcing thus the urgent need for intensive care and prophylactic treatment for cardiovascular diseases.

## Competing interests

The authors declare that they have no competing interests.

## Authors' contributions

FH (MD, associate professor of internal medicine and endocrinology) participated in the design of the study and drafting the manuscript. NF (MD, MPH) performed the statistical analysis and participated in the drafting the manuscript. DK (MD, MPH, PhD Candidate in Epidemiology) participated in the statistical analysis and revised the manuscript critically for important intellectual content. FS (MD, assistant professor of cardiology) and FA (MD, professor of internal medicine and endocrinology) participated in the design of the study and all authors have given final approval of the version to be published.
